# HIV incidence estimation among female sex workers in South Africa: a multiple methods analysis of cross-sectional survey data

**DOI:** 10.1016/S2352-3018(22)00201-6

**Published:** 2022-09-05

**Authors:** Reshma Kassanjee, Alex Welte, Kennedy Otwombe, Maya Jaffer, Minja Milovanovic, Khuthadzo Hlongwane, Adrian J Puren, Naomi Hill, Venice Mbowane, Kristin Dunkle, Glenda Gray, Fareed Abdullah, Rachel Jewkes, Jenny Coetzee

**Affiliations:** a Centre for Infectious Disease Epidemiology and Research, School of Public Health, University of Cape Town, Cape Town, South Africa; b The South African Department of Science and Innovation—National Research Foundation, Centre of Excellence in Epidemiological Modelling and Analysis, Stellenbosch University, Stellenbosch, South Africa; c Perinatal HIV Research Unit, Faculty of Health Sciences, University of the Witwatersrand, Johannesburg, South Africa; d School of Public Health, Faculty of Health Sciences, University of the Witwatersrand, Johannesburg, South Africa; e Wits Reproductive Health Institute, Faculty of Health Sciences, University of the Witwatersrand, Johannesburg, South Africa; f African Potential Management Consultancy, Kyalami, South Africa; g South African Medical Research Council, Cape Town, South Africa; h South African National Institute for Communicable Diseases, Johannesburg, South Africa

## Abstract

**Background:**

Although numerous studies have investigated HIV risk factors and shown high HIV prevalence among female sex workers in South Africa, no national HIV incidence estimate exists for this potentially important group for HIV transmission. We aimed to estimate HIV incidence among female sex workers in South Africa who could be accessed through sex worker programmes, and to refine and describe the methods that enabled analysis.

**Methods:**

This study was embedded in a cross-sectional national survey of female sex workers who were linked to sex worker programmes. We aimed to enrol 3000 female sex workers aged at least 18 years who had sold or transacted in sex in the preceding 6 months in 12 randomly selected districts of the 22 districts with sex worker programmes, ensuring coverage of all provinces of South Africa. Women who self-reported as current victims of human trafficking were excluded from enrolment. We used a multistep process to sample districts and then hotspots, and a chain referral method to recruit participants. We collected cross-sectional data for self-reported HIV status, demographic characteristics, and exposure to violence. Two rapid tests were used to ascertain diagnostic markers, a viral load assay was used to ascertain clinical markers, and the Maxim Limiting Antigen Avidity EIA was used to ascertain infection-staging HIV markers. Given the challenges of estimating HIV incidence, especially cross-sectionally, multiple methods of estimation were adapted to our setting, leveraging the age structure of HIV prevalence, recency-of -infection biomarker results (ie, where recent infection is classified as ≤1·5 normalised optical density [ODn] on the avidity assay and viral load of ≥1000 copies per mL), and reported testing histories.

**Findings:**

Of 3005 female sex workers who were enrolled and interviewed between Feb 4 and June 26, 2019, 2999 who had HIV test results were included in this analysis. The median age of participants was 32 years (IQR 27–38). 1714 (57·2%) of 2999 participants self-reported as being HIV positive, and 1447 (48·3%) of 2993 participants reported client sexual violence in the past year. The measured HIV prevalence was 62·1% (95% CI 60·3–65·7) and peaked at approximately age 40 years. Using recency-of-infection biomarker results, we obtained a base case estimate of HIV incidence of 4·60 cases per 100 person-years (95% CI 1·53–8·45) for the population. Estimates were generally consistent by method, and outlying incidence estimates calculated by self-reported testing histories were considered unreliable. Various sensitivity analyses produced estimates up to 11 cases per 100 person-years, and we did not detect differences by age and region.

**Interpretation:**

We found that female sex workers have extraordinarily high HIV incidence of approximately 5 cases per 100 person-years, emphasising the need to sustain and strengthen efforts to mitigate risk and provide adequate care. The notable role that sex work has in HIV transmission demands substantial investment in ongoing epidemiological monitoring.

**Funding:**

South African Medical Research Council, South African National Treasury, Global Fund, South African Department of Science and Innovation, Wellcome Trust.

## Introduction

Women are disproportionately affected by HIV in South Africa; of the 7·3 million adults living with HIV, 64% are women.^1^ A quarter of South African women aged 15–49 years are HIV positive.^1^ National incidence estimates for the general population suggest that the highest risk of acquisition is among 20–34-year-old Black African women, at 4·5% per year, declining steadily with age.^2^ This high prevalence and incidence of HIV is attributed to many factors, including age, concurrent partnerships, substance use, violence, and low level of education.^3^ HIV prevalence among female sex workers in South Africa is high, estimated to be 39–89% across different geolocations.^4^, ^5^ A national study of female sex workers reported a prevalence of 62%.^6^, ^7^ These data were collected before the COVID-19 pandemic, which is likely to have negatively affected provision of HIV services.

**Research in context**

**Evidence before this study**

Although many studies have observed high HIV prevalence among female sex workers in South Africa, a search of PubMed conducted before commencement of this study using the terms “female sex worker” AND “HIV” AND “incidence” AND “South Africa” for studies published in English between Jan 1, 2000, and March 31, 2019, showed that incidence had been observed only in small studies conducted in particular locales. More generally, South African women, especially adolescent girls and young women, have been shown to have high HIV incidence and prevalence. Absence of nationally representative incidence estimates for female sex workers has undermined efforts to quantify their needs as a vulnerable and highly affected population and their importance in the overall transmission of HIV. Without recognising the importance of this population, commitments are weakened to fund and sustain programmes aimed at supporting female sex workers at the national level. Cohort studies with vulnerable, mobile populations present notable difficulties to estimating incidence, including substantial exposure to bias. Previously described alternatives to cohort studies require nuanced context-appropriate adaptations, which are not yet widely understood and practised.

**Added value of this study**

We analysed data from the first national survey of female sex workers in South Africa, by deploying four logically distinct approaches to estimating HIV incidence. The primary finding is that, despite evidence of functioning support programmes for female sex workers with HIV, HIV incidence is sustained at about 5 cases per 100 person-years across a wide range of ages. To our knowledge, we report the first direct quantification of national HIV incidence among female sex workers who are accessible through sex worker programmes and provide a novel demonstration of multiple analytical approaches that are available to generate such estimates despite the methodological obstacles that are applicable to research among female sex workers (eg, scarce and cross-sectional data, small samples sizes, and little knowledge to inform analysis input parameters).

**Implications of all the available evidence**

Given that HIV incidence among female sex workers is high across a broad range of ages, sustained efforts are required to mitigate the risk of infection in this vulnerable population. The high incidence among female sex workers also means that clients of female sex workers have a high level of exposure to sexual contacts with newly acquired HIV, who are thus highly inectious, and supports the broader importance of female sex workers and their clients in the spread of HIV among the general population. Sustaining support programmes for female sex workers and expanding work to include risk mitigation for sex work clients and related research is crucial for sustained progress in HIV elimination.

Female sex work contributes greatly to transmission dynamics in HIV epidemics in sub-Saharan Africa. The high risk of HIV for female sex workers should be understood within the context of economic hardship, violence, and criminalisation of the profession.^4^, ^5^, ^8^, ^9^, ^10^ Modelling estimates suggest that less than 5% of new HIV infections in the country can be attributed to female sex workers; however, 42% of new infections are attributable to their male clients.^11^ Direct estimation of HIV incidence has been conducted only within small subgroups of the South African population of female sex workers.^12^ We are not aware of any nationally representative, direct estimates of HIV incidence among sex workers from any country in sub-Saharan Africa.^13^

In response to the challenges facing female sex workers, numerous HIV prevention programmes have been developed under the South African National Sex Workers Plan.^14^ Through these programmes, sex workers act as peer educators and provide outreach services, including commodity distribution, and facilitate access to primary health and psychosocial care through sex-worker-friendly service providers, which can immediately initiate pre-exposure prophylaxis and antiretroviral therapy. Reliable estimates of HIV incidence among female sex workers are crucial to understand the effect of preventive interventions, and HIV incidence is a primary outcome indicator of the National Sex Workers Plan. This plan aims to reduce new HIV, tuberculosis, and sexually transmitted infections; reduce mortality and morbidity through adequate care; and reach all vulnerable people.^14^

In 2019, the first South African national cross-sectional survey of female sex workers who were linked to sex worker programmes was conducted.^6^ Estimating incidence in these women is particularly challenging due to the cross-sectional nature of these data, small sample sizes for the relevant analyses, uncertain accuracy of self-reported data, and insufficient knowledge about this population to inform inputs that are required for incidence calculations.^15^, ^16^ By use of data and laboratory results from the national survey, we aimed to estimate HIV incidence among female sex workers in South Africa and to describe the method adaptations that we used to enable this analysis.

## Methods

### Study design and participants

Our study was embedded in a cross-sectional national survey of female sex workers who were linked to sex worker programmes, conducted in South Africa in 2019, and is described in detail in the study protocol.^6^ We conducted an analysis of the data from the national survey by use of multiple methods.

We aimed to enrol 3000 cisgender women aged at least 18 years, who had sold or transacted in sex in the preceding 6 months and worked in one of the districts that were studied. A multistep sampling process was followed.^6^ Briefly, a computerised simple random sampling procedure (SAS PROC SURVEYSELECT) selected 12 districts from the 22 eligible districts in South Africa that had active sex worker programmes (among 54 districts), stratified by province to ensure at least one district in each of South Africa's nine provinces was represented. Both provincial and district sample sizes were proportional to estimates of female sex-worker population size.^17^ A sample of mapped hotspots was drawn per district, ensuring representation across hotspot types (eg, brothel, tavern, or street). Seeds, defined as initial venue recruits, were initially identified by peer educators at sex work venues and, once enrolled by the sex work programmes, recruited subsequent female sex workers. Surveys were completed at the programme site or at their stations of sex work**.** A chain referral method was used for recruitment to enrol female sex workers, where every participant who was enrolled into the study was asked to distribute three coupons at random to fellow female sex workers. For ethical reasons, potential victims of human trafficking were excluded, and the relevant sex work programme was notified, enabling the provision of legal and psychosocial support.

Voluntary written informed consent was undertaken in a language of the participants choosing, from among the 11 official languages in South Africa, before data collection. This study and the questionnaire were approved by the University of the Witwatersrand Human Research Ethics Committee (reference number 180809).

### Procedures

A cross-sectional interviewer-administered survey was completed. The data captured included demographic characteristics, self-reported previous sexual history (eg, age at first sex and first commercial sex), client sexual violence (ie, during the past year), known HIV status, and previous treatment history. Whole blood samples were collected from all participants and couriered within 24 h, at a stable temperature, to a centralised district-level state laboratory dispatch, at which the samples were processed and refrigerated. From these National Health Laboratory Services laboratories, the samples were then couriered to the National Institute for Communicable Diseases incidence laboratory in Johannesburg, South Africa.

Parallel testing by two rapid HIV point-of-care tests was performed on each participant (from among ABON HIV 1/2/O Tri-Line HIV Rapid Test Device [ABON Biopharm, Hangzhou, China; 99·6% sensitivity and 100% specificity]; First Response HIV 1-2.O Card Test [Premier Medical Corporation, Gujarat, India; 99·2% sensitivity and 100% specificity]; and Toyo Anti-HIV 1/2 Test [Turk Lab, Izmir, Turkey; 99·2% sensitivity and 100% specificity]). Discordant results were resolved by a laboratory ELISA conducted by the National Health Laboratory Services.

Among participants who were HIV positive, viral load was quantified by use of 1 mL plasma on the COBAS AmpliPrep/COBAS TaqMan HIV-1 Test version 2.0 instrument (Roche Diagnostics, Mannheim, Germany) for automated extraction, amplification, and detection. The dynamic linear range of the assay is 20–107 copies per mL with dual-target detection of the gag and long terminal repeat regions to ensure broad genotype inclusivity.

The HIV-1 Limiting Antigen (LAg) Avidity EIA (Maxim Biomedical, Rockville, MD, USA; hereafter known as LAg Avidity EIA) was used to test for recent infection and provides a normalised optical density (ODn).^18^, ^19^

### Statistical analysis

All analyses were performed in R (version 3.6.3), with a combination of standard packages, including glm, and custom-developed code. Listwise deletion of missing values was performed, as, on review of the testing process, laboratory results could reasonably be assumed to be missing completely at random (≤5% missing). Data were generally analysed as is (ie, self-weighting), and without stratification due to the small sample size. For prevalence and incidence estimation, bootstrap percentile 95% CIs were calculated, with 50 000 bootstrap samples drawn to approximate the survey design (ie, sampling chains per district until district sample sizes were reached, then sampling people within chains).

**Figure 1:**
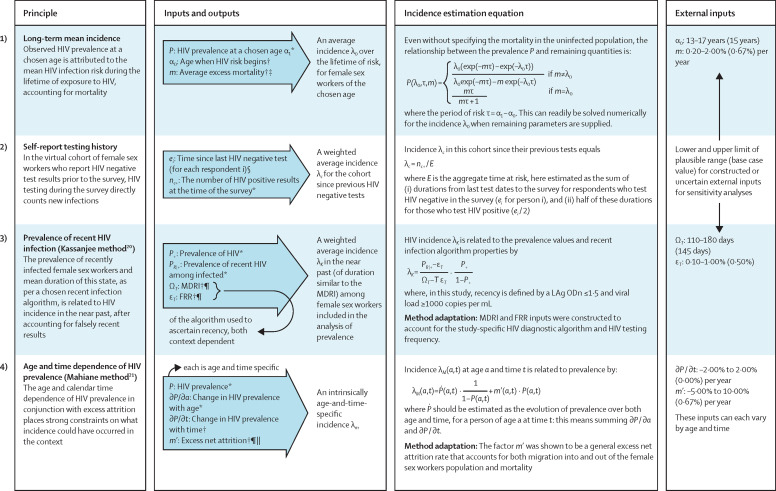
Methods of analysis used to estimate HIV incidence from the cross-sectional survey data For each method, the underlying principle, inputs and outputs, and central incidence estimation equation are specified, as well as method adaptations for this specific study setting. Ranges for external inputs for sensitivity analyses are also listed. Additional details are shown in the appendix (pp 1–17). FRR=false recent rate. MDRI=mean duration of recent infection. *Estimated directly from survey HIV data. †External inputs or assumptions. ‡Per-person mortality rate in people with HIV minus that in people without HIV. §Measured directly from survey self-reported HIV data. ¶Additional calculations performed to account for setting. ||Per person net outflow rate of people with HIV minus that of people without HIV.

Four logically independent incidence estimation approaches of varying sophistication were implemented, allowing for the comparison of results from methods with different advantages and challenges (figure 1). Where external inputs were required, base case inputs (ie, best estimates) were chosen or constructed on the basis of available knowledge, with low and high limits of plausible ranges considered in sensitivity analyses. All methods produced an estimate of the instantaneous incidence rate (expressed throughout as cases per 100 person-years), either occurring at the time of the survey or representing a weighted average during the months preceding the survey.

Methods are further described in the appendix (pp 1–17), which reports conceptual considerations, equation derivations, technical details on method adaptations for our setting, code made available with this work, and the choice of base case values and plausible ranges for inputs.

Most estimation methods require the prevalence of HIV as a function of age as an input, and method 3 requires the prevalence of recent infection by age. Prevalence by age was analysed by use of binomial regression, with each model form guided by statistical goodness-of-fit Akaike information criterion values and likelihood ratio tests.

Method 1 analysed long-term mean incidence. A rough estimate of mean historical HIV incidence was produced by considering a model world, defined to be one in which HIV incidence began abruptly at a chosen age and was constant for a predetermined period, while maintaining an excess mortality among individuals with HIV infection.

Method 2 used self-report testing history to analyse incidence. Female sex workers who self-reported their last HIV test as negative provided a virtual cohort of female sex workers, followed up from the negative test result to the survey, when new infections could be counted. To select women into the cohort, we considered a range of upper bounds for the time between the negative test and the survey date.

Method 3, or the Kassanjee method, used the prevalence of recent HIV infection. As described by Kassanjee and colleagues,^20^ this method required ascertaining recent infection among individuals who were HIV positive, by any chosen algorithm with sufficiently well estimated values for mean duration of recent infection (MDRI) and false recent rate (FRR). MDRI is the mean time after per-protocol detectable infection for which participants show the markers of recent infection, within some chosen time (*T*; typically 1–2 years) after infection (after which, recent infection would be considered spurious or false), ideally at least six months.^15^ FRR is the context-specific proportion of participants who are HIV positive who, despite being detectably infected for more than time *T*, nevertheless show the recent infection markers, ideally close to zero.^15^ In this method, HIV incidence was then estimated from the prevalence of HIV and recent HIV, and the MDRI and FRR.

For our analysis, we used a LAg ODn of 1·5 or less and a viral load of 1000 copies per mL or higher to identify recent HIV.^19^ The LAg Avidity EIA was used as the primary infection-staging test and produced an ODn.^19^ As per convention,^22^ ODn was dichotomised into high (ie, non-recent infection) and low (ie, recent infection) categories by the cutoff of 1·5.^23^ However, serological assays, such as variants on the LAg Avidity EIA, are highly prone to false recent results among individuals who are virally supressed,^22^ and a large FRR leads to imprecise and thus uninformative incidence estimates.^24^ Since many respondents who were HIV positive in this study were virally suppressed (1143 [64·4%] of 1774 participants had viral load ≤1000 copies per mL), mainly due to treatment (1453 [86·8%] of 1673 participants self-reported antiretroviral therapy use in an analysis of data from the same survey, which focused on the HIV cascade^7^), we adopted the widely used mitigation of including a viral load criterion in the definition of recent infection: cases with a viral load below a set threshold (ie, 1000 copies per mL) were defined as non-recent, independent of serological result.^25^ This mitigation reduced the FRR and, although often neglected, the applicable MDRI.

MDRI and FRR inputs were not readily available, as previous independent evaluations of a recent infection test based on the LAg Avidity EIA and viral load by the Consortium for the Evaluation and Performance of HIV Incidence Assays excluded data from people on treatment.^23^, ^25^ The results are, therefore, not directly generalisable to settings in which a substantial portion of the population initiated antiretroviral therapy and achieved viral suppression within a few months of becoming infected.^22^, ^25^ MDRI (and FRR) estimates were, therefore, adapted for this analysis to account for estimated testing and treatment initiation rates inferred from the intensive follow-up in sex worker programmes, by use of a method that has not previously been proposed (appendix pp 8–10).^26^

The incidence estimates were obtained by three structurally distinct analyses: pooling all data into a single risk group, fitting prevalence of both HIV and recent infection as a function of age and inferring age-specific incidence, and estimating incidence by client sexual violence during the past year and in two groups of districts on the basis of their observed HIV prevalence. There were insufficient data to produce high statistical power for analyses by age or subgroups, which are therefore primarily illustrative.

Method 4, or the Mahiane method, used the age and time dependency of prevalence to estimate incidence. As explored by various groups^27^, ^28^, ^29^ and made formally rigorous by Mahiane and colleagues,^21^ the age and time dependence of HIV prevalence, read in conjunction with mortality estimates, places strong constraints on what incidence could have occurred in a given population. Particularly, it has been shown that age-specific and calendar-time-specific HIV incidence is related to HIV prevalence, the rate of change of HIV prevalence, and excess mortality, which are all also age-specific and time-specific.

Inspection of the Mahiane estimator shows that the factor that is typically referred to as excess mortality is only strictly such when there is no other inflow or outflow of individuals other than through death. In a simple susceptible–infected epidemiological model with in and out migration (appendix pp 12–13), the factor can be more generally interpreted as an excess net attrition rate (ie, the difference in the net attrition rate of individuals with HIV infection compared with susceptible individuals). The net attrition rate in each group is the net rate of movement out of the group, considering both movement in and out of sex work and mortality. Loosely informed by our data where possible, this excess net attrition rate was allowed to vary within a chosen plausible range.

The rate of change of prevalence should be estimated accounting for the evolution of prevalence both over age and time, that is, by adding how prevalence varies with age to how it varies with the passing of time. In this analysis, we estimated HIV prevalence and its dependence on age from our data. Since data were derived from a single cross-sectional survey, providing no information on the dependence of prevalence on the passing of time, sensitivity analyses were used to consider a plausible range of values for this term. Mindful of the uncertainty around inputs required for this incidence estimation approach, we chose to include only 20–30-year-old female sex workers for this method.

### Role of the funding source

The funder of the study had no role in study design, data collection, data analysis, data interpretation, or writing of the report.

## Results

Of 3005 female sex workers who were enrolled, 2999 had known HIV status and were included in the anaylsis. Participants were aged 18–64 years (table 1), with a median age of 32 years (IQR 27–38). We enrolled and interviewed participants between Feb 4 and June 26, 2019. The proportion of participants who self-reported HIV infection on enrolment was lower than the proportion who were HIV positive on testing. A fifth of participants were located in the two districts where the district-level HIV prevalence observed in our sample of female sex workers was less than 50%. Prevalence for these districts was 26% (96 of 377 participants) and 37% (95 of 260 participants) versus an aggregated prevalence of 51–86% (1671 of 2362 in aggregate) across the other ten districts.

**Table 1:** Descriptive characteristics of the female sex workers in the sample

		**Number (%)**
**Overall (n=2999 with recorded HIV status)**		
Age, years (n=2999)		
	18–24	399 (13·3%)
	25–29	670 (22·3%)
	30–34	724 (24·1%)
	35–39	586 (19·5%)
	40–49	524 (17·5%)
	50–64	96 (3·2%)
HIV status as per testing (n=2999)		
	HIV positive	1862 (62·1%)
	HIV negative	1137 (37·9%)
Self-reported HIV status (n=2999)		
	HIV positive	1714 (57·2%)
	HIV negative	1254 (41·8%)
	Unknown	31 (1·0%)
HIV prevalence in district (n=2999)		
	Low prevalence (<50%)	637 (21·2%)
	High prevalence (≥50%)	2362 (78·8%)
Sexual client violence in the past year* (n=2993)		
	Yes	1447 (48·3%)
	No	1546 (51·7%)
**Self-report as HIV negative (n=1254)**		
Time from test to interview (n=1174)		
	<1 month	241 (20·5%)
	≥1 month and <3 months	411 (35·0%)
	≥3 months and <6 months	224 (19·1%)
	≥6 months and <1 year	157 (13·4%)
	≥ 1 year and <2 years	80 (6·8%)
	≥2 years	61 (5·2%)
HIV status as per testing (n=1254)		
	Positive	122 (9·7%)
	Negative	1132 (90·3%)
**HIV positive (n=1862)**		
LAg ODn (n=1796)		
	ODn ≤1·5	190 (10·6%)
	ODn >1·5	1606 (89·4%)
Viral load in copies per mL (n=1774)		
	Viral load <1000 copies per mL	1143 (64·4%)
	Viral load ≥1000 copies per mL	631 (35·6%)
Recent algorithm classification (n=1765)		
	Recent†	28 (1·6%)
	Non-recent	1737 (98·4%)

LAg=Maxim Limiting Antigen ELISA. ODn=normalised optical density.

* The respondent reported being forced to have sex or having sex because threatened by or afraid of client.

† LAg ODn ≤1·5 and viral load ≥1000 copies per mL.

Among female sex workers who self-reported as HIV negative, the median time since the last test was 78 days (IQR 36–188), and 1033 (88%) of 1174 had tested in the past year. Among female sex workers with HIV infection, the median LAg ODn was 3·9 (IQR 2·8–4·7). The median viral load was 18 100 copies per mL (IQR 4746–58 350).

**Figure 2:**
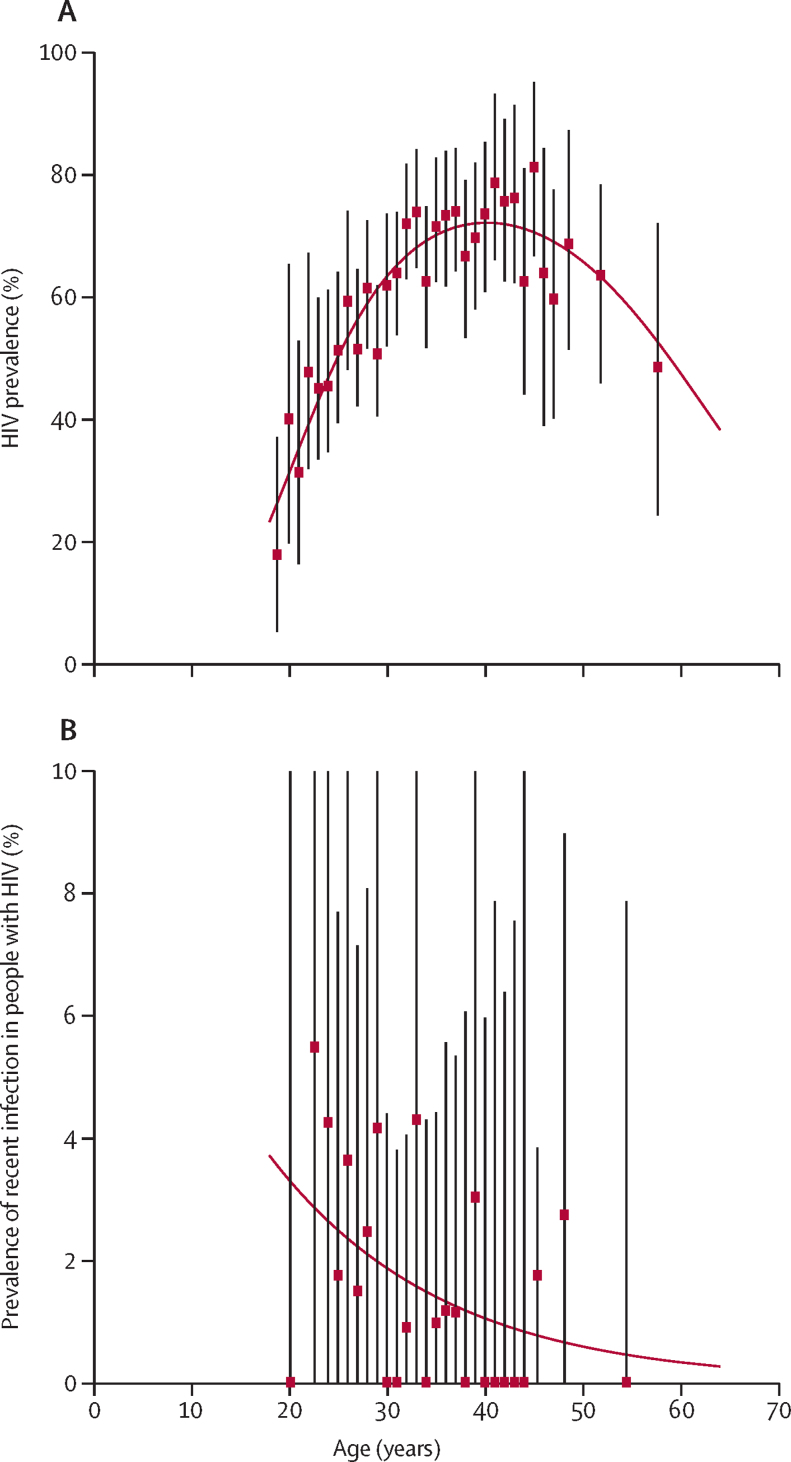
Age dependence of prevalence The prevalence of HIV among all respondents (A) and the prevalence of recent infection among respondents who were HIV positive (B). Prevalence point estimates and bootstrap percentile CI limits are indicated by markers and whiskers, adjacent whole ages are grouped to ensure n>30 per estimate. Smoothed curves were obtained from fitting a binomial regression for prevalence (logit link) by use of cubic polynomial of age (A) or linear function of age (B) as predictors.

Overall HIV prevalence (62·1%, 95% CI 60·3–65·7) peaked at approximately 40 years (figure 2), and the prevalence of recent infection (identified by LAg ODn ≤1·5 and viral load of ≥1000 copies per mL) among individuals who were HIV positive was small across ages (overall 1·6%, 95% CI 0·9–2·4).

Collated incidence estimates (table 2) show that three of the four methods yielded similar base case incidence estimates of 4–7 cases per 100 person-years for the overall sample of female sex workers, and for these methods, estimates reached up to 11 cases per 100 person-years in sensitivity analyses. We were unable to discern differences in incidence by age, region, and client violence. The estimate relying solely on self-report data (method 2) was about three times higher than others.

**Table 2:** Incidence estimates based on the multiple analysis methods by subset of female sex workers and scenario or inputs

		**Incidence estimate, cases per 100 person-years**	**95% CI**
**Method 1: long-term mean incidence**			
Hypothetical cohort now aged 30 years			
	Base case	6·88	..
	Sensitivity analysis: varying age of sexual debut, excess mortality, and HIV prevalence	5·46–9·59	..
**Method 2: self-report testing history**			
Individuals who self-report last known test results as negative, and provide the dates of the test			
	Base case: previous test within 5 years of survey	21·65	17·10–29·35
	Sensitivity analysis: previous test within 1–4 years of survey	21·37–27·36	..
**Method 3: prevalence of recent HIV infection (Kassanjee method)**			
All female sex workers			
	Base case	4·60	1·53–8·45
	Sensitivity analysis: extreme mean duration of recent infection or false recent rate inputs	2·55–6·16	..
Aged 20–30 years			
	Base case, including only respondents of specified ages in analysis	6·09	1·83–12·71
Aged 20–40 years			
	Base case, including only respondents of specified ages in analysis	5·25	1·98–10·03
Aged 20 years			
	Base case, using all data to perform age-structured analysis	5·17	0·90–16·97
Aged 30 years			
	Base case, using all data to perform age-structured analysis	5·65	1·83–10·99
In districts with HIV prevalence ≥50%			
	Base case, including only respondents belonging to specified group in analysis	5·80	1·42–11·19
In districts with HIV prevalence <50%			
	Base case, including only respondents belonging to specified group in analysis	3·02	0·00–8·57
Client sexual violence in the past year			
	Base case, including only respondents belonging to specified group in analysis	5·47	0·96–13·03
No client sexual violence in the past year			
	Base case, including only respondents belonging to specified group in analysis	3·85	0·00–8·23
**Method 4: age and time dependence of prevalence (Mahiane method)**			
Aged 20 years			
	Base case	4·68	1·65–7·24
Aged 30 years			
	Base case	4·83	1·70–7·48
	Sensitivity analysis: varying uncertain input for rate of change of prevalence with time in plausible range	0·00–9·82	..
	Sensitivity analysis: varying uncertain input for excess attrition in plausible range	1·43–10·42	..

**Figure 3:**
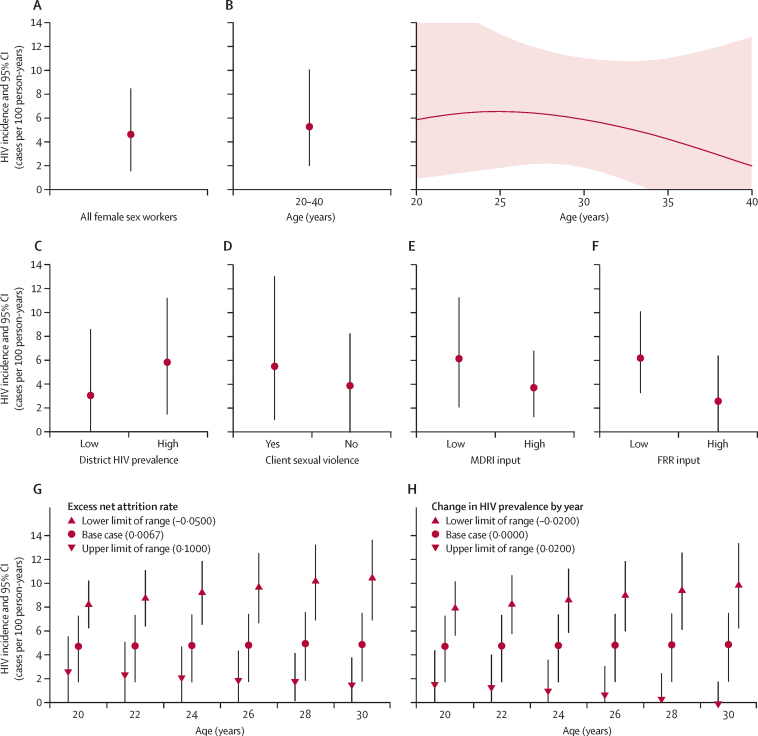
HIV incidence estimates Overall estimate (A), age disaggregation (B), estimate by high or low HIV prevalence in districts (C), estimate by occurrence of client sexual violence (D), estimate by use of extremal plausible mean duration of recent infection estimates (E), and estimate by use of extremal plausible false recent rate estimates (F), obtained by use of recent HIV-infection data. Estimates showing sensitivity to estimates of the excess net attrition rate (per year) of sex workers with HIV infection (G) and showing sensitivity to estimates of the time gradient of prevalence (per year) among sex workers (H), obtained by use of age or time dependence of HIV prevalence. The smoothed curve by age in (B) was obtained from fitting binomial regressions (logit link) for HIV prevalence by use of of a quadratic function of age and for recent infection by use of a linear function of age. MDRI=mean duration of recent infection. FRR=false recent rate.

For scenarios with base-case inputs, the estimate reported is the point estimate for incidence. For sensitivity analyses that vary inputs, the estimate reported is the range of point estimates obtained. 95% CIs are not reported for method 1, given the simplistic assumptions made for this method, or for sensitivity analyses. More detail on sensitivity analyses results is given elsewhere (figure 3, appendix pp 3–5). Values for inputs are given elsewhere (figure 1, appendix pp 2–15).

Results from individual methods are briefly summarised here; further details are available in the appendix (pp 1–17). For method 1, a mean lifetime incidence of 5·46–9·59 cases per 100 person-years is needed to realise the regression-based HIV prevalence of 63·3% (95% CI 60·7–67·7) among female sex workers aged 30 years, by use of different inputs.

In the virtual cohort of female sex workers who self-reported previous HIV negative tests (method 2), the outlying incidence estimate was 21·37–27·36 cases per 100 person-years with different data inclusion rules.

By use of the information contained in the recent infection data (method 3), the overall HIV incidence estimate was 4·6 cases per 100 person-years using base case inputs. Uncertainty overshadowed any age-dependency of incidence (figure 3), as was the case when we compared incidence in low-prevalence and high-prevalence districts (p=0·39) and by report of client sexual violence in the past year (p=0·49). Incidence estimates range from 2·55 to 6·16 cases per 100 person-years when varying MDRI and FRR inputs (figure 3).

When making inferences about female sex workers aged 20–30 years by use of age and time dependency of HIV prevalence (method 4), the input for age dependence of HIV prevalence was directly estimated from the data (figure 2). However, there was high uncertainty in the remaining two crucial age-specific inputs required, namely, estimates of the applicable excess net attrition rate and the rate of change of prevalence as a function of calendar time. Our implementation of this method thus focused on understanding the range of intrinsically age-dependent incidence estimates obtained by varying these two inputs. The base case incidence estimates are similar by age; however, estimates varied substantially with inputs (figure 3). It is also possible that some plausible variation of excess attrition by age (or, less expectedly, variation in the time dependence of HIV prevalence by age) would imply an age trend in incidence that we are unable to identify with confidence in this analysis.

## Discussion

To our knowledge, this study offers the first estimate of HIV incidence among female sex workers in South Africa who are linked to sex worker programmes at a national level. Incidence was estimated to be in the region of 4·60–6·88 cases per 100 person-years and was supported by three of four methods (excluding the self-report method). Our analysis shows how different methods can be used to gain useful insights into incidence rates from cross-sectional data by leveraging the age structure of HIV prevalence and recency-of-infection biomarker results.

The high HIV incidence estimated in our study is not unprecedented in surveys and has been seen repeatedly in the era before widespread testing and treatment, including in general population contexts among adolescent girls and young women (ie, aged 15–24 years; 5·5 cases of HIV per 100 person-years, 95% CI 4·5–6·5)^30^ and pregnant women (10·7 cases of HIV per 100 person-years, 8·2–13·1).^3^ A cohort study of women from eThekwini, South Africa, who had been raped and opened a police case were reported to have an incidence of 6·6 cases of HIV per 100 person-years (95% CI 4·8–9·1) compared with 4·7 cases of HIV per 100 person-years (3·5–6·2) in the control group.^31^ Incidence was also recorded at 4·4 cases of HIV per 100 person-years (95% CI 3·2–5·8) in the placebo group of a prevention trial among women in an African multisite study, which included sites in South Africa.^32^ Although we would like to see a much lower HIV incidence among sex workers, it would be a mistake not to regard our present estimates as indicative of successes in sex work programmes, especially given that the HIV incidence among sex worker populations is not substantially higher than the HIV incidence found in some general populations discussed here.

In our study, there were no discernible differences in incidence by age or region. The extraordinarily high risk of HIV acquisition is only partly offset, in terms of net harm, by high treatment coverage with moderate viral suppression rates. Given that female sex workers, by definition, have multiple sexual partners (and engage in various high-risk sexual activities),^5^ sustained high incidence has implications for transmission beyond the study population, with our findings providing empirical support for modelling estimates that 41·9% of new infections are attributable to clients of sex workers who bridge to the general population.^11^ Furthermore, our findings, supported by additional analyses of our sample,^7^ emphasise the need for extensive efforts to improve viral suppression in female sex workers in supporting South Africa to achieve the UN 95–95–95 targets, including in this population. The high risk of HIV for sex workers and potential for onward transmission lend further credence to sex work continuing to be epidemiologically significant to the HIV pandemic and suggests the importance of interventions targeting client populations. However, the SARS-CoV-2 pandemic and resultant lockdown interventions are already having severe effects on economically vulnerable groups and the health system in general. We are concerned that the broadly positive cascade of care indicators that are observed among populations of female sex workers who are linked to sex worker programmes are under threat and will need to be reviewed.

Estimating HIV incidence at the population level presents multiple challenges to which there are no fully satisfactory solutions. In this analysis, multiple methods of estimation were investigated. Notably, the simple long-term mean incidence method obtained similar results to the nominally more sophisticated incidence estimates that were produced by the Kassanjee method^20^ and the Mahiane method.^21^ This work provides considerable practical detail beyond that contained in the seminal papers deriving the general relations on which these formal methods are based. We show new ideas that are relevant to the contextual adaptation of the properties of recency-of-infection tests that are required in the Kassanjee method and a derivation for the expanded notion of excess net attrition rate, which generalises the more narrowly defined excess mortality of the Mahiane method. The analysis that was based on data for self-reported testing history produced an outlying high incidence estimate. This estimate is likely to be unreliable and might be due to a social desirability bias in reporting, such as notable under-reporting of the time since a negative test or spurious reporting of previous negative tests. A limitation of the analysis was the need to resort to several sensitivity analyses, in the absence of robust estimates of key inputs. However, this need underlines the inherent challenges that affect all attempts to obtain reliable incidence estimates from cross-sectional survey data: the difficulty of obtaining robust data for treatment, mortality, and migration; the importance of local adaptation of estimates of recent infection test properties; and the reality that it is preferable to have data from multiple surveys, of substantial size, conducted a few years apart. We would advise that all analyses based on these core ideas of recent infection and age or time structure of prevalence should include sensitivity analyses. Due efforts should be made to obtain the best possible characterisation of the survey population to optimise accuracy of external inputs and avoid overly large ranges of plausible values. Point estimates produced by the Kassanjee method were reasonably insensitive (2·55–6·16 cases per 100 person-years), relative to uncertainty in the base case estimate, to variations in locally derived recency-of-infection test properties, based on available knowledge about the recency-of-infection test and the testing and treatment behaviour of the survey population. In our analysis, the Mahiane estimates were particularly vulnerable to gaps in our knowledge of the age-specific inputs about attrition and temporal trends in HIV prevalence.

Statistical uncertainties around estimates were large due to the sample size. Furthermore, differentiating incidence by age or discrete groups, such as regions or type of sex work, would require either substantially larger samples, such as in AIDS Indicator Surveys and Population HIV Impact Assessment surveys, or the comparison of groups with large incidence differences. With a larger sample, estimates from the Kassanjee and Mahiane methods could be combined into an optimally weighted average. Both approaches would benefit from future work to optimise the efficiency of the estimators for a complex survey design, which we accounted for using an ad-hoc bootstrapping approach that imitated the sampling process. Our estimates also relate specifically to female sex workers who were accessible through sex worker programmes, and although participants were encouraged to recruit others at random, we might not have achieved a completely random or representative sample.

Our study provides a baseline measure against which ongoing HIV programme efficacy can be measured. The importance of sex worker-associated HIV transmission demands substantial investment in ongoing epidemiological monitoring, including future large surveys similar to the one reported here. Although there is evidence of a decline in incidence in some high-risk groups, including adolescent girls and young women,^30^, ^33^ sex workers are still at high risk of HIV infection. This level of risk is despite broad gains in HIV programmes targeting the population and offering access to pre-exposure prophylaxis and rapid antiretroviral therapy initiation, emphasising the need to strengthen efforts, including to provide access to pre-exposure prophylaxis across age groups. Although these gains might have been affected by the COVID-19 pandemic, there is an urgent need to improve understanding and intervention against the risk of bridging populations. Clients of sex workers not only present the primary risk of infection to female sex workers but also the risk of onward transmission in the general population, and no programmes exist to target this key population in South Africa. The failure to address the primary risk population for female sex workers will decrease the ability of programmes to reduce the risk of new infections in female sex workers.

## Data sharing

Deidentified individual participant data that underlie the results in this Article are available at https://data.mendeley.com/datasets/rpv326nkvg. Existing public tools are available for implementing the primary incidence estimation methods (R library inctools), and R code for any novel adaptation to this setting has been published alongside this Article at https://github.com/rkassanjee/mdri-fta, as described in the appendix.

## Declaration of interests

We declare no competing interests.
