# Supplementary material for: HIV incidence estimation among female sex workers in South Africa: a multiple methods analysis of cross-sectional survey data
Source: Lancet HIV. 2022 Sep 5;9(11):e781–90. doi: 10.1016/S2352-3018(22)00201-6 (PMC9626386; doi:10.1016/S2352-3018(22)00201-6)
Supplement: Supplementary appendix [file mmc1.pdf]

THE LANCET
HIV

Supplementary appendix

This appendix formed part of the original submission and has been peer reviewed. We post it as supplied by the authors.

Supplement to: Kassanjee R, Welte A, Otwombe K, et al. HIV incidence estimation among female sex workers in South Africa: a multiple methods analysis of cross-sectional survey data. *Lancet HIV* 2022; published online Sept 5. https://doi.org/10.1016/S2352-3018(22)00201-6.

Supplementary Material to

HIV incidence estimation among female sex workers in South Africa: a multiple methods analysis of cross-sectional survey data

by Kassanjee et al, Lancet HIV 2022

Introduction

This appendix supplements the main presentation of our HIV incidence analysis from the first nationally representative survey of (street/hotspot based) female sex workers in South Africa. It provides further conceptual and technical details to our calculations.

We emphasize that the estimation of HIV incidence remains ultimately very challenging and problematic for various reasons, especially when working with limited data such as a single cross-sectional dataset. This holds even when the dataset is large and of high quality, as we regard the present one to be – the dataset includes, among many other details, explicit age structure, background information on testing histories, and ascertainment of 'recent' infection according to previously characterised stable biomarkers.

Hence, the purpose of documenting these technical details is not to stage a spirited defence of their theoretical optimality or universal robustness, but to

- support basic transparency of the analysis which, with limitations freely noted, informs our view that HIV incidence is i) in the region of 5% per annum, and ii) consistently at this high level across a wide age range.
- inform replication and adaptation of these analyses to similar data.

That said, we doubt that there is much scope for a more refined incidence analysis, given inherent limitations in survey data of this kind, even though the present data set is substantial both in detail and the number of respondents.

Contents

Method 1: Long term mean incidence estimation 2
Method 2: Self-report testing history 5
Method 3: Prevalence of 'recent' HIV infection (Kassanjee method) 6
Method 4: Age / time dependence of prevalence (Mahiane method) 12
Other notes 16
References 17

Method 1: Long term mean incidence estimation

Method 1 summary

In this method, the observed HIV prevalence, at some chosen age, is attributed to the lifetime exposure to HIV infection risk.

In particular, a rough estimate of 'mean' historical HIV incidence can be produced by considering a model world in which HIV incidence begins abruptly at some age (a_0) and then maintains a constant value (λ_0) for some period (τ), all the while maintaining an 'excess mortality' amongst HIV infected individuals of m – the excess mortality is the additional mortality rate experienced by HIV infected, compared to uninfected, persons, in absolute terms. Even without specifying the mortality in the uninfected population, it can be shown that the relationship between the quantities described and prevalence (P) after the period of risk is:

$$P(\lambda_0, \tau, m) = \begin{cases} \dfrac{\lambda_0 \left(exp(-m\tau) - exp(-\lambda_0\tau)\right)}{\lambda_0\, exp(-m\tau) \; - \; m\, exp(-\lambda_0\tau)} & \text{if } m \neq \lambda_0 \\ \dfrac{m\tau}{m\tau + 1} & \text{if } m = \lambda_0 \end{cases}$$

This relation can readily be solved, numerically, for the incidence λ_0, when all the other parameters are supplied.

A simple model to relate prevalence, incidence and time

The key to 'Method 1' in the main manuscript is a simple model of constant incidence in an unstructured cohort of individuals with a known initial prevalence P_0, further simplified in our case by setting P_0 to zero at age 15.

Consider a cohort of initially S_0 susceptible individuals suddenly entering an infection risk category at time $t = 0$, with risk of infection per unit time $\lambda = \lambda_0$. Let the mortality of susceptible individuals be μ and the mortality of infected individuals be $\mu + m$. In the language of ordinary differential equations, we can represent the rule for the variation, over time, of the susceptible and infected populations ($S(t)$ and $I(t)$) as

$$\frac{dS}{dt} = -(\lambda + \mu)S$$

$$\frac{dI}{dt} = \lambda S - (\mu + m)I$$

It is straightforward to solve that, after a time τ spent at risk, the remaining susceptible population is given by

$$S(\tau) = \mathrm{S_0}\exp(-(\lambda + \mu)\tau).$$

Substituting this into $\frac{dI}{dt}$ gives, after some standard manipulations

$$I(\tau) = \mathrm{S_0}\frac{\lambda}{m - \lambda}\, \exp(-(\mu + \mathrm{m})\tau)\;\; [\exp(-(\lambda - \mathrm{m})\tau)\;\; - 1]$$

If we worry that we are in the (not physically plausible) limit that $m = \lambda$ holds *precisely*, in which case the denominator becomes zero, the whole expression can be simplified to

$$I(\tau) = \mathrm{S_0}\, \lambda\, \tau \exp(-(\mu + \mathrm{m})\tau).$$

The prevalence of infection $P(\tau)$ is defined by

$$P(\tau) = \frac{I(\tau)}{I(\tau) + S(\tau)}$$

and we can substitute the above solutions into $S(\tau)$ and $I(\tau)$ to obtain

$$P(\lambda,\tau,m) = \begin{cases} \dfrac{\lambda\left(exp(-m\tau) - exp(-\lambda\tau)\right)}{\lambda\, exp(-m\tau) \; - \; m\, exp(-\lambda\tau)} & \text{if } m \neq \lambda \\ \dfrac{m\tau}{m\tau + 1} & \text{if } m = \lambda \end{cases}$$

as indicated in the manuscript. It seems worth highlighting that, as is also a key feature of the more general analysis of Mahiane et al,[1] the baseline mortality μ drops out of the calculation, and we can effectively model this process completely (for the purpose of relating incidence to prevalence and time) by just incorporating the 'excess mortality' (m) experienced in the infected state.

We map this dynamical model onto our cohort of respondents by thinking of λ as the *mean* incidence experienced from age 15, without extreme variations, for about 20 years. We know that incidence is not likely to be constant for 20 years of life, for a cohort drawn from the population at large, but for a group moving in and out of sex work this may not be unreasonable, since the risk is plausibly driven by the sex work itself more than by other time/age varying contacts. Bear in mind that the local population-level prevalence (and thus likely the prevalence among clients) has been on something like a high plateau for about 20 years.

We then use our regression of prevalence on age (Figure 2, Part A, of manuscript) to estimate the cumulative effect of incidence from age 15 to a somewhat arbitrary age at which it seems we have a statistically robust prevalence estimate. In particular, we chose age 30 as the reference age as it is near the middle of the range of respondents' ages, where the prevalence estimate was robust to details of choice in regression (smoothing) method employed. We considered variations of the age of sexual debut (13 to 17 years).

As noted previously, we are not claiming that this simple model provides a refined view of the prevalence/incidence/mortality process over 15-20 years, but it offers a way to interpret the fact that prevalence at an age of around 30-35 years is well estimable from data as we have, and this must reflect a long-term trade-off of incidence and (excess) mortality in a cohort reaching this age. There is a complex averaging implied here, which we do not propose to unpack in great detail.

Obtaining prevalence as a function of age from the survey data

A core element of the female sex worker (FSW) survey data set is the HIV status observations, which, although they occurred over a period of some months, we regard as having occurred at roughly a single point in time, and which are each labelled by the age of a participant. It is straightforward and routine to assume/specify a plausible parametric form to capture the prevalence as a function of age, or to apply a 'black box' smoothing routine. For this analysis, we fitted the serostatus data by binomial regression (logit link) on a cubic polynomial of age (Figure 2, Part A of manuscript) – providing an estimated prevalence at age 30 to be 63% (95% CI: 61%,68%).

Estimating the excess mortality

The simple model underpinning this method requires an estimate of the mean prevailing 'excess mortality' associated with infection. We need to estimate this for HIV positive sex workers, relative to HIV negative sex workers, for the age group 15-30, as applicable over the last 15 years, for most of which ART has been readily available, at least in principle. Expressed in units of deaths 'per year', an excess mortality of 0.1 (i.e. 10% per annum), on top of a baseline of no mortality, would imply a life expectancy of 10 years post infection. At present, because persons on stable HIV treatment have so little excess mortality, it is difficult to estimate it. While the evidence base is ever evolving, and not without controversy, we propose, for exploratory purposes, a plausible range for the excess HIV mortality is 0.2-2% p.a (with the lower and upper limits roughly 3-fold lower and higher than the base case estimate used of 0.67%). This spans an order of magnitude, with the maximum value in the range implying a 50-year post-infection life expectancy if there were no baseline mortality.

Incidence estimates

In Table 1 below, the 'high HIV incidence' scenario is obtained by setting each of the inputs (excess mortality, age of sexual debut, and HIV prevalence) to the maximum values in the chosen ranges, since higher values of any of these inputs would result in higher incidence estimates. The 'low HIV incidence' scenario is obtained by setting each input to the minimum value in the range considered. For HIV prevalence, the point estimate and 95% CI limits of regression-based estimates for prevalence at age 30 are used for the base case and high and low incidence scenarios, respectively.

Table 1. Incidence estimates based on mean incidence experienced from age of sexual debut (varied) to age 30, which would lead to the survey-estimated prevalence at age 30, given an excess mortality post infection (varied)

**Scenario**	**Inputs**			**Incidence estimate (% p.a.)**
	**Excess mortality (% p.a)**	**Age of sexual debut (years)**	**HIV prevalence (%)[1]**	
**Base case**	0·67	15	63	6·88
**High HIV incidence**	2·00	17	68	9·59
**Low HIV incidence**	0·20	13	60	5·46

Per annum, p.a.

Method 2: Self-report testing history

Method 2 summary

This method considers incidence in a virtual cohort of FSWs who self-reported their most recent previous HIV tests to be negative.

The incidence (λ_c) experienced by this cohort, since their previous tests, equals

$$\lambda_c = n_{c+}/E$$

where n_{c+} is the number of FSWs who test HIV-positive at their interviews (having reported that their most recent previous HIV test was negative), and E is the aggregate time at risk, which, In this analysis, was estimated as the sum of (i) durations from self-reported test dates to the survey interviews for respondents who test HIV-negative in the survey, and (ii) half these durations for those who test HIV-positive.

Data inclusion

If the data on testing histories were entirely reliable, and the incidence was essentially constant for some period H preceding the survey, then it would be uncontroversial to apply the proposed 'Method 2' of the manuscript. The method measures the incidence among respondents who were unaware of their infection status at the time of the survey but were able to report a well specified most recent negative test. For consistency, one would calculate the exposure time among respondents who had a most recent negative test more recently than a time H into the past, with something like a midpoint or exponential infection date estimation (it matters little, uncertainty being dominated by the small number of infections) for those who test positive in the survey. Confronted with a real-world dataset that no doubt reflects biases of various kinds – the severity of which is likely to vary with the data inclusion criteria – it is not obvious how best to proceed. We considered a range of inclusion criteria: most strictly that we consider respondents whose most recent negative tests were less than one year before the survey; and most leniently considering all respondents who reported having a previous negative test no more than 5 years before the survey. There turned out to be no choice of this cut-off for which the HIV incidence estimate corresponded reasonably with the estimates obtained using the other three methods presented. It therefore does not appear that there is a satisfactory way of focusing on a reliable subset of testing history reports.

Incidence estimates

Table 2. Incidence estimates for a virtual cohort defined by self-report of negative last previous tests, varying the maximum allowed time from this test to the survey date

**Maximum time from negative test to survey (years)**	**Counts of FSWs**		**Aggregate time at risk (years)**	**Incidence (% p.a.)**	
	**Total**	**HIV positive at survey**		**Estimate**	**95% CI**
**1**	1033	69	252	27·36	20·1,38·3
**2**	1113	84	352	23·87	17·8,32·6
**3**	1150	99	429	23·09	18·0,31·3
**4**	1165	102	477	21·37	16·4,29·1
**5**	1174	109	503	21·65	17·1,29·4

Confidence interval, CI; Female Sex Workers, FSWs; Human Immunodeficiency Virus, HIV; Per annum, p.a.

Method 3: Prevalence of 'recent' HIV infection (Kassanjee method)

Method 3 summary

This analysis proceeds according to the previously described method of Kassanjee et al,[2] which requires ascertaining 'recent' infection amongst those who are HIV infected, by any chosen algorithm with sufficiently well estimated values for the following:

- Mean Duration of Recent Infection (**MDRI**): The average time, post per-protocol detectable infection, for which subjects exhibit the markers of 'recent' infection, within some chosen time (T) after infection (after which 'recent' infection would be considered spurious, or 'false') – typically ideally at least half a year,[3] and
- False Recent Rate (**FRR**): The context-specific proportion of HIV positive subjects who, despite being detectably infected for more than time (T), nevertheless exhibit the 'recent' infection markers – ideally close to zero.[3]

In this method, HIV incidence (λ_k) is then related to the prevalence of HIV (P_+) and prevalence of 'recent' infection amongst HIV-positive individuals ($P_{R|+}$) by

$$\lambda_K = \frac{P_{R|+} - \varepsilon_T}{\Omega_\mathrm{T} - T\,\varepsilon_T} \cdot \frac{P_+}{1 - P_+}$$

where Ω_T is the MDRI and ε_T is the FRR associated with the time cut-off T.

In the present work, the Maxim Limiting Antigen (LAg) Avidity EIA assay[4,5] was used as the primary infection staging test. The normalised optical density (ODn) produced is conventionally dichotomised into low ODn ('recent' infection) and high ODn ('non-recent') categories by the choice of a threshold, typically in the region of 1·5.[6] However, it is well known that serological assays such as variants on the LAg assay are highly prone to 'false recent' results among virally suppressed individuals.[7,8] This is problematic as a large FRR leads to uninformative (imprecise) incidence estimates (not biased estimates, as often erroneously believed).[9] Since the majority of HIV positive respondents in this study were virally suppressed (64·5% had VLs less 1000 copies per ml) mainly due to treatment, we therefore adopted the widely used mitigation of including a viral load (VL) criterion in the definition of 'recent' infection: we defined cases with a VL below a set threshold as 'non-recent', independent of serological result.[7] This dramatically reduced the FRR, although it is often neglected that this also reduces the applicable MDRI since some subjects naturally have low VLs between infection and the time cut-off T. The particular combination of thresholds on the serological assay (for the present analysis, ODn 1.5) and the VL assay (1000 copies per ml) which were used to define 'recent' infection must then be used to estimate the applicable MDRI and FRR.

The possibility of a significant portion of the population initiating ART and achieving viral suppression within a few months of becoming infected was explicitly excluded in previous recency test evaluations (for examples, see CEPHIA results articles[7,8]). Published MDRI estimates were thus adapted to account for the locally estimated testing and treatment initiation rates inferred from the intensive follow up of women known to the SW support programmes, using a method not previously proposed.

The Kassanjee incidence estimates were obtained by three structurally distinct analyses: 1) pooling all data into a single risk group; and 2) fitting both prevalence, as well as the prevalence of 'recent' infection among positive subjects, as a function of age, and inferring age-specific incidence. While a number of group comparisons were considered, sample sizes do not support such disaggregation of the data – for illustration, 3) incidence estimates are shown by a dichotomized district-level HIV prevalence and whether the client reported experiencing sexual client violence in the last year.

General remarks about Kassanjee estimator

There are numerous references which provide various detailed observations on the concepts, nuances, and potential interpretations attendant on the use of the Kassanjee estimator, which forms the basis of 'Method 3' of the manuscript – not least of all the seminal paper in which it is derived.[2,9] For convenience and coherence of the present arguments, we repeat here some useful heuristics and other key points.

- A test for recent infection is any reproducible algorithm for assigning, to a subject with confirmed HIV infection, a status of either 'recent' infection or 'non-recent infection', as long as it has the properties that:
    1. The probability of being classified as 'recent' is close to 1 for times soon after infection, and
    2. The probability of being classified as 'recent' is close to 0 for times long after infection.

- There is no 'gold standard' for a definition of recent infection, and hence no standard use for terminology such as 'sensitivity', 'specificity', and 'predictive values'.

- A recent infection test has two crucial performance characteristics, which, if defined and estimated carefully, capture all that needs to be known about the test in its application to incidence estimation using cross-sectional data:

    1. **The Mean Duration of Recent Infection (MDRI)** – *the mean time, post detectable infection, for which individuals exhibit the state of 'recent' infection defined by the test, within some specified 'time cut-off T post detectable infection (with T realistically chosen to be somewhere between 6 and 30 months).*

       This captures the biology of the specific biomarker evolution post infection, though it may have an element of (hopefully minor) contextual dependence through such factors as prevalent subtype mix and the applicable case definition of 'detectable infection'. For the Kassanjee approach to work optimally, the MDRI should be estimable from a small number of strong studies done separately from, and well before, any particular survey application. In our main article, we confront a limitation of this generalisability of existing MDRI estimates that is posed by a high frequency of early treatment in our study population, which impacts the intra-individual progression of HIV associated biomarkers. A useful rule of thumb is that a test yielding an MDRI of less than several months is unlikely to provide meaningfully precise HIV incidence estimates, given actual incidence rates and feasible survey sample sizes.

    2. **The False Recent Rate (FRR)** – *the probability that an arbitrarily chosen person, in a specified context, among available individuals who have been (detectably) infected for more than the specified 'time cut off' T, will yield a result of 'recent' infection.*

       This unavoidably captures a complex combination of biological *and* contextual factors, including maturity of the epidemic and treatment coverage, and so it should always be the focus of some careful analysis. An FRR value that is not comfortably below 1% poses a significant loss in precision of incidence estimates, and opens the door to potentially severe biases resulting from problems with its estimation. These statistical errors and biases are amplified by high HIV prevalence.

- Incidence estimates should always be analysed for sensitivity to the available test property estimates (MDRI and FRR). We particularly caution against a practice of declaring the FRR 'sufficiently close' to zero that it can be 'assumed to be' zero.

- The effort required to define and characterise a test for recent infection should not be under-estimated. Unless a research project accesses extensive resources and specific expertise, tests should not be developed 'on the fly'. More reasonably, values of 'thresholds'[‡] may be varied for particular applications, as long as these are based on previously validated assays/biomarkers. This may provide some level of adaptation/optimisation to unusual contexts.

- Optimisation of an assay/biomarker can be made precise by considering the minimisation of the standard error in a proposed survey design, performed in a specified context.[9] Notwithstanding some misconceptions to the contrary – there is no meaningful inherent 'bias' in incidence estimates which can be laid at the door of particular biomarkers, or the Kassanjee analysis per se. Biases in the analysis arise from *incorrect estimation* of the properties of the assay, while the actual/true biomarker dynamics, no matter what they are, merely place (often severe) limits on the possible precision of incidence estimates which can be generated.

MDRI Estimation

It is tempting, but problematic, to treat estimates of the MDRI as numbers that can be found on some sort of 'package insert' for a previously validated recency test. There are several subtleties.

In this study, the previously validated Limiting Antigen (LAg) assay formed the basis of the recent infection testing algorithm, with measurements below some threshold interpreted as identifying 'recent' infections. Below, we discuss how we identified relevant published MDRI estimates, and adapted these estimates to obtain MDRI inputs relevant to our setting.

Adjustment for viral load criterion

We know from previous analyses that the main complication of treatment is that immune response markers, such as obtained by the LAg assay, experience attenuated growth, and even 'revert' to low values, in subjects receiving effective treatment. This is the reason that the definition of recent infection is adapted to include more than just the LAg ELISA optical density – so that a 'sufficiently low' viral load (defined by some practically informative threshold), independent of LAg ODn (normalised optical density), is interpreted as a sufficient criterion for 'non recent' infection.

Some years ago, when early treatment was unusual, this refinement of the recent infection test algorithm mainly served to identify and reclassify otherwise 'false' recent results (i.e. results in people who were infected long enough – longer than the cut off time T introduced above – that they should have been classified as 'non-recent', but who, on account of ARV-induced viral suppression, look 'recent' on the serology alone).

In practice, of course, subjects may have low viral loads at any time, including at times soon after infection, though this is less common. Nevertheless, previous analyses have already noted how introducing a viral load criterion affects the MDRI, even when there is no early treatment, just because there is a background of low viral load results found among people soon after becoming detectably infected. This effect is incorporated in previous MDRI estimates from the CEPHIA[†] consortium[7] and so we need merely choose a LAg ODn threshold and a viral load threshold to find the appropriate 'base' MDRI in the literature (for our specified cut-off T defined above), to which we then applied the 'screening assay' and 'frequent testing' adjustments described below.

[‡] This refers to the thresholds typically used in translating continuous assay/biomarker measurements (e.g. of the immune response) into the dichotomous 'recent' or 'non-recent' classifications, as part of the test for recent infection algorithm. For example, in the current work, the algorithm adopted classifies as 'recently' infected those individuals with a Limiting Antigen Assay measurement below its threshold of 1·5 ODn (normalised optical density) *and* a viral load measurement above its threshold of 1000 copies/ml.

[†] CEPHIA: *The Consortium for the Evaluation and Performance of HIV Incidence Assays*. CEPHIA was established to independently assess promising biomarkers for algorithms for recent infection, and has provided international leadership and guidance on the use of this incidence approach. For a review of CEPHIA, see Murphy et al, Epidemiology and Infection, 2016.

Adjustment for HIV diagnostic screening assay

Testing for recent infection, logically, only occurs among individuals who are already considered to be confirmed as infected. It is well known that there are significant differences between the analytical sensitivity / detection thresholds of various diagnostic assays and markers of HIV infection. It makes sense to interpret these differences mainly as differences in 'time to detectable infection' post infectious exposure.[10]

This also complicates the estimation of MDRI using specimens pooled across multiple studies. For this reason, the CEPHIA collaboration[3] engaged in substantial data curation to harmonise the estimation of 'time since infection' for specimens from different studies which were assembled into a repository. The key insight is that while the precise data of infection is seldom known, it is feasible to locate estimates of 'time since' or 'relative to' an arbitrary but well-defined event. In many studies, this has, for example, been taken to be time relative to the date on which there is a fully developed Western Blot result, as that was for many years a standard definition of a 'confirmed' infection, and that is the choice reflected, for example, in estimates from the CEPHIA consortium, which we are recycling here.[7,8] This means that in tables of estimates of the MDRI, presented by assay and threshold, numbers from the CEPHIA collaboration[7] should be interpreted as the hypothetical MDRI which would be realised if subjects were 'confirmed' to be positive by having a fully developed western blot.

In the present work, diagnosis was performed by the use of two modern point of care rapid tests, which we estimate to show seroconversion about 6 days before a full western blot.[11] In the present study, we also used the Maxim Limiting Antigen (LAg) Avidity assay, which has a somewhat longer MDRI (at the standard threshold ODn =1·5) than the more well-known Sedia LAg assay.[5] Beginning with the published CEPHIA MDRI estimate (see supplementary content to the CEPHIA results article[7]) relevant to the recent infection testing algorithm employed in this study, after adjusting for the diagnostic assay and specific form of the Limiting Antigen assay, we obtained a rounded MDRI estimate of 180 days (before further adjustment for frequent testing, described below).

Frequent testing adaptation

As mentioned above, under general remarks about the Kassanjee incidence estimation framework, it is highly preferable for at least the MDRI of a recency test to be amenable to infrequent estimation/update – on the assumption that it captures mainly stable biological and contextual factors, and not factors which vary from time to time. However, the reality is that the studies on which existing estimates of MDRI are based were conducted on subjects who primarily did not receive very early treatment. While specimens from recipients of early testing were available, they: i) were a significant minority, ii) represent a not very clearly specified range of timings of treatment initiation, and iii) have been excluded from the MDRI estimation analyses of which we are aware. In the present FSW survey, there is reason to believe (based on logs and reports of the FSW support programmes that formed the partners for identifying 'hotspots' for this study) that there is very frequent testing, followed, in the case of diagnosis, by rapid treatment initiation.

We therefore now demonstrate a simple analysis which incorporates these ideas into a rough adjustment of the MDRI under the influence of frequent testing and rapid initiation. This may not withstand detailed cross examination, but it will provide us with some intuition for the size of the effect.

The standard way that the MDRI (Ω_T) is estimated is by

1. fitting biomarker data from seroconverters to extract an estimate of the function $P_R(t)$, which captures the probability that an arbitrarily selected person, from those available who have been (detectably) infected for time t, will yield a 'recent' test result, and then,
2. recalling the recency time cut-off T introduced earlier, measuring the area under this curve, between $t = 0$ and $t = T$.

Formally,

$$\Omega_T = \int_0^T P_R(t)\, dt.$$

For the present analysis, we referred to previous data/analyses for an estimate of $P_R(t)$, when there is no rapid diagnosis and treatment initiation. The form of $P_R(t)$ used is consistent with the MDRI noted above of 180 days.

To incorporate frequent testing, we define the probability of being artificially virally suppressed by treatment, as a function of time since infection, as $P_{VS}(t)$ and we remark that the new probability of testing 'recent' $P_R'(t)$ now becomes the probability that the subject

1. is *not* artificially virally suppressed (probability $1 - P_{VS}(t)$)
   AND
2. also tests 'recent' according to the recent infection testing algorithm (assumed to remain the probability $P_R(t)$ above).

We can write

$$P_R'(t) = \left(1 - P_{VS}(t)\right) P_R(t)$$

and calculate the MDRI $\Omega_T{}'$ from

$$\Omega_T' = \int_0^T P'_R(t)\, dt.$$

Using 'expert input' from the field from two of the authors (Maya Jaffer and Jennifer Coetzee, who are involved in liaison with FSW support programmes) we proposed a particular form of the testing and suppression dynamics which translates into our rough estimate of $P_{VS}(t)$ and thus our MDRI adjustment. $P_{VS}(t)$ was constructed assuming that

- 35% of FSWs never test.
- 20% of FSWs test regularly every 3 months.
- 45% of FSWs test less frequently, at a constant event rate of on once per year.
- The distribution of times from testing to viral suppression is a Weibull distribution defined by a 50th percentile of 2.5 months and 98th percentile of 6 months (also see [12,13]).

This process led to a base case MDRI estimate of 145 days. For sensitivity analyses, we consider extreme MDRI inputs of 110 days (obtained by constructing $P_{VS}(t)$ based on our most optimistic expert opinion insights into rapid testing and suppression) and 180 days (the MDRI that would erroneously be used were one to neglect accounting for frequent testing).

An implementation of this method for calculating a tailored MDRI Ω_T', encompassing greater flexibility in the construction of $P_{VS}(t)$ than employed above, has been deployed in R.[14]

FRR Estimation

As noted above, even a base estimate for the FRR for a recency test is not as simple as a number found in a published table, as is the often hoped to be the case more closely for the MDRI. The preceding discussion already confronted a number of challenges in adapting the available MDRI estimates into one that is applicable to the current protocol and context. While the factors affecting the FRR are in principle even more complex, there is also a key simplification; namely that the incidence estimation approach becomes less useful when it is dependent on a high precision (small relative standard error) of the FRR estimate. We instead mainly need to confirm that there is little risk of the FRR being too high (heuristically, too high means in the region of about 1%) and then we can consider a plausible range in a sensitivity analysis.

Treatment coverage is very high in the study population, and viral suppression is highly prevalent among treated individuals, as confirmed in the current dataset and analysed separately as part of the treatment cascade assessment. This suggests that the majority of long-infected individuals are virally suppressed, and effectively flagged, by this viral suppression, as 'non-recent' infections, given the case definition of 'recent' infection.

False-recent results from untreated individuals are mainly driven by (for example, see CEPHIA results[7,8]):

- elite controllers, who are rare, and themselves do not universally test 'recent' (rather around 30%), and
- a tail of slow biomarker progressors who are neither elite controllers, nor on treatment at times 2-4 years after infection – and the evidence suggests these are also rare.

Furthermore, the CEPHIA analysis[7] of FRRs constructed standard scenarios in which the emergent FRR values were calculated, given the available estimates of $P_R(t)$ and the frequency of false-recent results in key subsets of long infected people such as elite controllers and those on treatment.

We read the above as all providing evidence of the plausible range of FRR values for our algorithm and population being between 0·1% and 1%.

Fitting the prevalence of HIV and 'recent' HIV as functions of age

The common application of the Kassanjee analysis has been to estimate, in a surveyed population,

- a single HIV prevalence, and
- a single prevalence of 'recent' infection among HIV positives.

There is no reason, other than a shortage of data, not to fit both these prevalence values as functions of any combinations of predictors, either continuous or discrete, and thus also estimate incidence as functions of those predictors. See, for example [15].

In the present case, we fitted these prevalence values as functions of age. We fitted each prevalence by standard binomial regression with a logit link function, using quadratic and linear polynomials in age for the prevalence of HIV and the prevalence of recency among HIV positives, respectively.

Method 4: Age / time dependence of prevalence (Mahiane method)

Method 4 summary

As explored by various groups[16-19] and made formally rigorous by Mahiane et al,[1] the age and time dependence of prevalence, read in conjunction with mortality estimates, places strong constraints on what the incidence could have occurred in the context that produced this data.

The key result of the Mahiane analysis is that incidence $\lambda_M(a,t)$ at a given age a and time t, is related to prevalence by:

$$\lambda_M(a,t) \;=\; \dot{P}(a,t)\cdot\frac{1}{1-P(a,t)} + m'(a,t)\cdot P(a,t)$$

where $P(a,t)$ is the age and time dependent prevalence, $\dot{P}(a,t)$ its rate of its change in the population being studied and $m'(a,t)$ is the similarly dependent 'excess mortality' in HIV-positive individuals, compared to HIV-negative individuals.

Technically, the rate of change of prevalence $\dot{P}(a,t)$ should be estimated noting the evolution of prevalence both over age and time, for a person of age a at time t. This means adding how prevalence varies with age ($\partial P/\partial a$) to how it varies with the passing of time ($\partial P/\partial t$). In the present case, where data was derived from a single, cross-sectional survey, there is no explicit data on the dependence of prevalence on the passing of time. As there was significant age dependence of prevalence in our study, and, at the current time in South Africa, not much calendar-time dependence on prevalence,[20-22] the bulk of the contribution will come from the age dependence, which was estimated from the data. We consider a sensitivity analysis based on a plausible range of values for 'rate of change of prevalence by calendar time'. Considering these aspects, we also chose to only include an age range of 20-30 years, inclusive, for this approach.

A close inspection of the derivation of the Mahiane estimator[1] reveals that the factor m', typically referred to as 'excess mortality', is only strictly an excess mortality in the case when there is no other in or outflow of individuals other than through death. In a simple susceptible/infected (SI) epidemiological model with in and out migration, it can be shown that the factor m' finds interpretation in a more general sense as an 'excess net attrition rate' of infected, relative to susceptible, individuals – i.e. equals the net (per capita) outflow for infected persons minus that for susceptible persons.

General remarks about the Mahiane Estimator

Though originally presented via a different derivation, the Mahiane estimation procedure[1] used in 'Method 4' can also be understood as a direct generalisation of the model of 'Method 1' above, stripped of all special assumptions about either i) the prevalence at some reference age (or time) or ii) constancy of any mortality or incidence over periods of time or ranges of age.

The estimator can be derived by starting with the same equations we introduced for Method 1 above:

$$\frac{dS}{dt} \;=\; -(\lambda+\mu)S$$

$$\frac{dI}{dt} \;=\; \lambda S-(\mu+m)I$$

where the symbols have the same meanings as before, but we consider the more general case where it is understood that all the state variables (S and I) and parameters (λ,μ and m) are allowed to vary arbitrarily as functions of age and time. We need not introduce partial derivatives here if we understand the total time derivatives to be taken

with respect to *time passing from the point of view of a cohort of individuals sharing a given age*, and we can later unpack this according to

$$\frac{d}{dt} = \frac{\partial}{\partial t} + \frac{\partial}{\partial a}.$$

The partial time and age derivatives of the prevalence are estimated by whatever means are available. In the present case, we directly estimate the age dependence from the survey data, and consider, by way of sensitivity analysis, a plausible range for the calendar time dependence, given the quasi-stable prevalence seen in South Africa in recent years.

The derivation of the core Mahiane estimator proceeds not by solving the model equations, in contrast to Method 1, as this is not possible under the more general conditions now being considered. The key point is that we can directly evaluate

$$\frac{dP}{dt} = \frac{d}{dt}\left(\frac{I}{I+S}\right) = (1-P)(\lambda - mP).$$

Note once again the disappearance of the mortality of the susceptible group. This can be rearranged into the basic Mahiane estimator:

$$\lambda = \frac{I}{1-P}\frac{dP}{dt} + mP.$$

The important generalisation we want to consider is to accommodate migration, so we incorporate both in and out migration into the basic SI model:

$$\frac{dS}{dt} = -(\lambda + \mu)S + \alpha S$$

$$\frac{dI}{dt} = \lambda S - (\mu + m)I + \beta I$$

where α and β are rates which are chosen to express the *net population inflows*, other than through infection and death, in proportion to the S and I populations.

To express this in a form structurally the same as the original form, we can define

$$\mu' = \mu - \alpha$$

and

$$m' = m - \beta + \alpha.$$

The SI model equations, with migration, become

$$\frac{dS}{dt} = -(\lambda + \mu')S$$

$$\frac{dI}{dt} = \lambda S - (\mu' + m')I.$$

It follows that

$$\lambda = \frac{I}{1-P}\frac{dP}{dt} + m'P$$

and so incidence estimation follows the same prevalence-fitting process as before, as in Mahiane et al,[1] with the additional task of estimating the newly defined m'. m' is now a general **excess net attrition rate** (i.e. the net per capita outflow among infected persons minus the net per capita outflow among susceptible persons).

Estimating the Excess Net Attrition rate

For Method 4, we considered 20-30 year old FSWs only, in part to allow for more informed, albeit still uncertain, selection of plausible ranges of unknown inputs.

Base estimate

In the absence of migration, the 'excess net attrition rate' of infected individuals is just the excess mortality as defined for Method 1.

Adjustment for recruitment of new sex workers, and departure from sex work

In the presence of migration, the 'excess net attrition rate' of infected individuals is the sum of i) excess mortality, as defined above, and ii) excess net attrition from all other sources – combining the movements of persons into and out of sex work. In the absence of insights into movements *out of* sex work obtainable from the current dataset, and differing views by researchers on whether this rate is expected to be higher amongst HIV positive or HIV negative FSWs, we focused on understanding the contributions to the latter term from movements *into* sex work.

A basic component of the Mahiane analysis is that, in the simple case, there is no movement of individuals between, into and out of categories except through infection and death. If new population members are recruited (which in a whole-population analysis would mean conventional immigration), then it matters whether the inbound population comes with the same prevalence as the established population.

In the present case, the data suggests that the prevalence of FSWs who have recently begun sex work is not distinguishably different from the prevalence among those who have been active in sex work for some time. We plotted HIV prevalence (with uncertainties) as a function of *time since starting sex work* (recorded in whole years), and, by eye, found that HIV prevalence as the time in sex work approached zero was similar to HIV prevalence in the overall group (52%). This also held when separately considering 20-25-year-olds (prevalence of 45%) and 26-30-year-olds (57%).

A lower prevalence in those entering sex work would have suggested a positive contribution to the excess net attrition rate term, and higher prevalence a negative contribution; with the magnitude of the contribution increasing with greater differences in prevalence values and a larger rate of entry into sex work.

Although our simple analysis did not provide strong evidence for the sign of the base case contribution, based on the observed entry rate (25% of FSWs reported being in sex work for at most 2-3 years), and a sensitivity analysis that allowed the prevalence of those entering sex work to differ by ~5% (either direction) from those already in sex work, we chose a wide plausible range for the overall 'excess net attrition rate' of -5% to 10%.

Adjustment for calendar-time dependence of prevalence

As there was significant age dependence of prevalence in our study, and, at the current time in South Africa, not much calendar-time dependence on prevalence, for our analysis subset of 20-30 year old FSWs, the bulk of the contribution will come from the age dependence, which was estimated from the data. That is, the partial age derivative of prevalence, which we obtain directly from data, will be much larger than the partial time derivative.

We thus considered a sensitivity analysis based on a plausible range of values for 'rate of change of prevalence by calendar time', which we varied from -2% to 2%.

Fitting Prevalence as a function of age

We begin by considering the case of *incidence* not being meaningfully distinguishable by age, but prevalence depending sharply on age, though hardly (in recent years) on time. We can build this into a standard regression of prevalence by age, by fitting

$$\ln(1 - P(a)) = A - \Lambda a$$

which captures these points, with Λ being the prevailing incidence if there is no mortality, $P(a)$ the HIV prevalence at age a , and A and Λ the regression model parameters.

We can generalise the conditions of our estimate by interpreting Λ as

$$\frac{1}{1-P}\frac{\partial P}{\partial a}$$

and then obtaining more appropriate incidence estimates via

$$\lambda = \frac{1}{1-P}\frac{dP}{dt} + m'P = \Lambda + \frac{1}{1-P}\frac{\partial P}{\partial t} + m'P$$

with $\frac{\partial P}{\partial t}$ and m' estimated/varied through a plausible range, as proposed above.

In fitting this regression to our dataset, criteria such as AIC values and likelihood ratio tests do not justify adding higher order terms to the regression of prevalence on age or using alternative transformations of prevalence.

From our data, we were able to estimate Λ as 4·42 (95% CI: 1·32,7·04), which was then the estimate of HIV incidence in the absence of a time-dependence of prevalence and in the case of a zero excess net attrition rate.

Importantly, when including inputs for the time-dependence of prevalence and the excess net attrition rate, to obtain incidence estimates, both these inputs can be expected to vary by age and time, and thus the inputs add additional age-dependencies to incidence estimates.

Other notes

Comparison of results when applying methods to the FSW survey data

While the analyses underlying the three methods that do not rely on self-reported data are logically different, in this work, they are all implemented using data from a single survey. Conventional statistical confidence intervals incorporate the effect of reproducibility between surveys. Hence, to the extent that there are no significant differences in bias between methods, we expect them to agree better than implied merely by the confidence interval sizes, which they do (see manuscript for results). This agreement suggests that there are no surprising substantial differences between the three methods.

Weighted averages of estimates

With a larger sample, estimates from the Kassanjee and Mahiane methods could be combined into an optimally weighted average.[15]

References

1. Mahiane GS, Ouifki R, Brand H, Delva W, Welte A. A general HIV incidence inference scheme based on likelihood of individual level data and a population renewal equation. *PLOS ONE* 2012. **7**(9): e44377.
2. Kassanjee R, McWalter TA, Barnighausen T, Welte A. A new general biomarker-based incidence estimator. *Epidemiology* 2012. **23**(5):721-728.
3. Murphy G, Pilcher CD, Keating SM, et al. Moving towards a reliable HIV incidence test - current status, resources available, future directions and challenges ahead. *Epidemiol Infect* 2017. **145**(5):925-941.
4. Duong YT, Qiu MF, De AK, et al. Detection of recent HIV-1 infection using a new Limiting-Antigen Avidity Assay: Potential for HIV-1 incidence estimates and avidity maturation studies. *PLOS ONE* 2012. **7**(3):e33328.
5. Sempa JB, Welte A, Busch MP, et al. Performance comparison of the Maxim and Sedia Limiting Antigen Avidity assays for HIV incidence surveillance. *PLOS ONE* 2019. **14**(7):e0220345.
6. Duong YT, Kassanjee R, Welte A, et al. Recalibration of the limiting antigen avidity EIA to determine mean duration of recent infection in divergent HIV-1 subtypes. *PLoS One* 2015. **10**(2):e0114947.
7. Kassanjee R, Pilcher CD, Busch MP, et al. Viral load criteria and threshold optimization to improve HIV incidence assay characteristics. *AIDS* 2016. **30**(15):2361-2371.
8. Kassanjee R, Pilcher CD, Keating SM, et al. Independent assessment of candidate HIV incidence assays on specimens in the CEPHIA repository. *AIDS* 2014. **28**(16):2439-2449.
9. Kassanjee R, McWalter TA, Welte A. Short communication: Defining optimality of a test for recent infection for HIV incidence surveillance. *AIDS Res Hum Retroviruses* 2014. **30**(1):45-49.
10. Grebe E, Facente SN, Bingham J, et al. Interpreting HIV diagnostic histories into infection time estimates: analytical framework and online tool. *BMC Infect Dis* 2019. **19**(1):894.
11. Delaney KP, Hanson DL, Masciotra S, Ethridge SF, Wesolowski L, Owen SM. Time until emergence of HIV test reactivity following infection with HIV-1: Implications for interpreting test results and retesting after exposure. *Clin Infect Dis* 2017. **64**(1):53-59.
12. Gay CL, Mayo AJ, Mfalila CK, et al. Efficacy of NNRTI-based antiretroviral therapy initiated during acute HIV infection. *AIDS* 2011. **25**(7):941-949.
13. Myer L, Phillips TK, McIntyre JA, et al. HIV viraemia and mother-to-child transmission risk after antiretroviral therapy initiation in pregnancy in Cape Town, South Africa. *HIV Med* 2017. **18**(2):80-88.
14. Kassanjee R, Welte A. mdri-fta [Source code]. 2021. https://github.com/rkassanjee/mdri-fta.
15. Grebe E, Welte A, Johnson LF, et al. Population-level HIV incidence estimates using a combination of synthetic cohort and recency biomarker approaches in KwaZulu-Natal, South Africa. *PLOS ONE* 2018. **13**(9):e0203638.
16. Brookmeyer R, Konikoff J. Statistical considerations in determining HIV incidence from changes in HIV prevalence. *Statistical Communications in Infectious Diseases* 2011. **3**(1).
17. Brunet RC, Struchiner CJ. A non-parametric method for the reconstruction of age- and time-dependent incidence from the prevalence data of irreversible diseases with differential mortality. *Theor Popul Biol* 1999. **56**(1):76-90.
18. Hallett TB, Zaba B, Todd J, et al. Estimating incidence from prevalence in generalised HIV epidemics: Methods and validation. *Plos Medicine* 2008. **5**(4):611-622.
19. Williams B, Gouws E, Wilkinson D, Karim SA. Estimating HIV incidence rates from age prevalence data in epidemic situations. *Stat Med* 2001. **20**(13):2003-2016.
20. Simbayi L, Zuma K, Zungu N, et al. South African National HIV Prevalence, Incidence, Behaviour and Communication Survey, 2017. Cape Town: HSRC Press 2019.
21. UNAIDS. Country factsheets South Africa 2020. https://www.unaids.org/en/regionscountries/countries/southafrica (accessed 14 August 2021).
22. Shisana O, Rehle T, Simbayi LC, et al. South African National HIV Prevalence, Incidence and Behaviour Survey, 2012. Cape Town: HSRC Press 2014.
